# A double-blind, placebo-controlled, randomized trial of PXT3003 for the treatment of Charcot–Marie–Tooth type 1A

**DOI:** 10.1186/s13023-021-02040-8

**Published:** 2021-10-16

**Authors:** Shahram Attarian, Peter Young, Thomas H. Brannagan, David Adams, Philip Van Damme, Florian P. Thomas, Carlos Casanovas, Jafar Kafaie, Céline Tard, Maggie C. Walter, Yann Péréon, David Walk, Amro Stino, Marianne de Visser, Camiel Verhamme, Anthony Amato, Gregory Carter, Laurent Magy, Jeffrey M. Statland, Kevin Felice

**Affiliations:** 1 Reference Center for Neuromuscular Disorders and ALS, CHU La Timone, Marseille, France; 2 Department of Neurology, Medical Park Bad Feilnbach, Bad Feilnbach, Germany; 3 Columbia University Medical Center, The Neurological Institute, New York, USA; 4 French Reference Center for Rare Peripheral Neuropathies, Service de Neurologie Adulte, APHP, CHU Bicêtre, Le Kremlin Bicêtre, France; 5 Department of Neurology, University Hospitals Leuven, KU, Leuven, Belgium; 6 Center for Brain & Disease Research, VIB, Leuven, Belgium; 7 Department of Neurology, Hackensack University Medical Center, Hackensack, USA; 8 Department of Neurology, Saint Louis University School of Medicine, St. Louis, USA; 9 Neuromuscular Unit, Neurology Department, Bellvitge University Hospital, Barcelona, Spain; 10 Neurometabolic Diseases Group, Bellvitge Research Institute (IDIBELL) and CIBERER, Barcelona, Spain; 11 U1171, Centre de référence des maladies neuromusculaires Nord Est Ile de France, Hôpital Salengro CHU de Lille, Lille, France; 12 Department of Neurology, Friedrich-Baur-Institute, Ludwig-Maximilians-University of Munich, Munich, Germany; 13 Centre de Référence Maladies Neuromusculaires AOC, Filnemus, Euro-NMD, CHU Nantes, Hôtel-Dieu, Nantes, France; 14 Clinical Neuroscience Research Unit, University of Minnesota, Minneapolis, USA; 15 University of Michigan Health System, Ann Arbor, MI USA; 16 Department of Neurology, Amsterdam University Medical Centres, University of Amsterdam, Amsterdam Neuroscience, Amsterdam, The Netherlands; 17 Department of Neurology, Brigham and Women’s Hospital, Boston, USA; 18 St. Luke’s Rehabilitation Institute, Physical Medicine and Rehabilitation, Spokane, USA; 19 CHU Dupuytren, Limoges, France; 20 University of Kansas Medical Center, Kansas City, USA; 21 Department of Neuromuscular Medicine, Hospital for Special Care, New Britain, USA

**Keywords:** Charcot–Marie–Tooth, CMT1A, Neuromuscular disorder, Overall Neuropathy Limitations Scale, PMP22, PXT3003, Randomized controlled trial

## Abstract

**Background:**

Charcot–Marie–Tooth disease type 1A (CMT1A) is a rare, orphan, hereditary neuromuscular disorder with no cure and for which only symptomatic treatment is currently available. A previous phase 2 trial has shown preliminary evidence of efficacy for PXT3003 in treating CMT1A. This phase 3, international, randomized, double-blind, placebo-controlled study further investigated the efficacy and safety of high- or low-dose PXT3003 (baclofen/naltrexone/D-sorbitol [mg]: 6/0.70/210 or 3/0.35/105) in treating subjects with mild to moderate CMT1A.

**Methods:**

In this study, 323 subjects with mild-to-moderate CMT1A were randomly assigned in a 1:1:1 ratio to receive 5 mL of high- or low-dose PXT3003, or placebo, orally twice daily for up to 15 months. Efficacy was assessed using the change in Overall Neuropathy Limitations Scale total score from baseline to months 12 and 15 (primary endpoint). Secondary endpoints included the 10-m walk test and other assessments. The high-dose group was discontinued early due to unexpected crystal formation in the high-dose formulation, which resulted in an unanticipated high discontinuation rate, overall and especially in the high-dose group. The statistical analysis plan was adapted to account for the large amount of missing data before database lock, and a modified full analysis set was used in the main analyses. Two sensitivity analyses were performed to check the interpretation based on the use of the modified full analysis set.

**Results:**

High-dose PXT3003 demonstrated significant improvement in the Overall Neuropathy Limitations Scale total score vs placebo (mean difference: − 0.37 points; 97.5% CI [− 0.68 to − 0.06]; *p* = 0.008), and consistent treatment effects were shown in the sensitivity analyses. Both PXT3003 doses were safe and well-tolerated.

**Conclusion:**

The high-dose group demonstrated a statistically significant improvement in the primary endpoint and a good safety profile. Overall, high-dose PXT3003 is a promising treatment option for patients with Charcot–Marie–Tooth disease type 1A.

**Supplementary Information:**

The online version contains supplementary material available at 10.1186/s13023-021-02040-8.

## Background

Charcot–Marie–Tooth disease is a group of rare, hereditary, chronic and debilitating diseases of the peripheral nerves that result first in weakening and atrophy of the foot and leg muscles as well as the hand and arm muscles, then in distal sensory loss and, in some patients, limb deformity [1]. Patients suffer from progressive reduced mobility and fine motor skills that all impact quality of life [2]. There is no cure; current disease management options are limited to symptomatic treatment, such as medications for pain and fatigue, the use of support measures, such as ankle/foot orthoses and arm crutches, and corrective surgery [3, 4].

The most common form of the disease, Charcot–Marie–Tooth type 1A (CMT1A), originates in the duplication of the peripheral myelin protein (*PMP22*) gene located in chromosome 17p11.2, which results in overexpression of *PMP22*, a key component of the myelin sheath produced by Schwann cells. An abnormally high level of *PMP22* protein leads to altered structure and dysfunction of the myelin sheath and causes demyelination, reduced motor nerve conduction velocities (< 38 m/s), and length-dependent axonal loss [3, 5]. Therefore, regulating *PMP22* expression could be a plausible treatment for CMT1A [3]. Past clinical trials of pharmacologic agents aimed at reducing *PMP22* expression were unsuccessful: ascorbic acid did not improve patient condition [6], and the progesterone receptor antagonist onapristone was not safe in humans [3]. However, a combination of baclofen, naltrexone and D-sorbitol in low doses (PXT3003) could be effective in treating CMT1A as each agent interferes with *PMP22* expression differently. Baclofen, a γ-aminobutyric acid (GABA)-B receptor agonist prescribed to relieve spasticity, acts via the cyclic adenosine monophosphate (cAMP)-dependent silencer element to negatively regulate *PMP22* transcription in Schwann cells [7, 8]. Low doses (0.1 mg/kg) of naltrexone, an opioid antagonist used to treat opiate and alcohol dependence, raise endogenous endorphin release [9], increase cell surface targeting of cognate opioid receptors [10], and are expected to potentiate the adenylate cyclase-inhibiting effect of endogenous opioids on the level of *PMP22* expression. D-Sorbitol, a carbohydrate alcohol used as a sweetener in the food industry (E420) and as a laxative, can bind with high affinity to muscarinic receptors [11, 12] that are involved in *PMP22* expression, and possibly improve protein folding [13, 14]. Pre-clinical studies showed that PXT3003 limits *PMP22* production, alleviates abnormal Schwann cell differentiation and improves neuromuscular function [15–17].

A subsequent double-blind, placebo-controlled phase 2 clinical trial involving 80 subjects with mild to moderate CMT1A investigated the efficacy and safety of three PXT3003 doses (highest baclofen/naltrexone/D-sorbitol daily dose [mg]: 6/0.7/210). The study demonstrated the safety and tolerability of PXT3003 and provided preliminary evidence of efficacy for the highest dose [18]. Because the efficacy of PXT3003 increased with dose, higher doses of PXT3003 were anticipated to have greater efficacy while maintaining a good safety profile.

Following the results of the phase 2 trial, a multi-center, randomized, double-blind, placebo-controlled phase 3 study was conducted exploring the efficacy and safety of two PXT3003 doses (baclofen/naltrexone/D-sorbitol [mg]: 6/0.70/210 and 3/0.35/105) administered twice daily compared to placebo in 323 subjects diagnosed with mild to moderate CMT1A. The results of the phase 3 study are reported here.

## Results

**Fig. 1:**
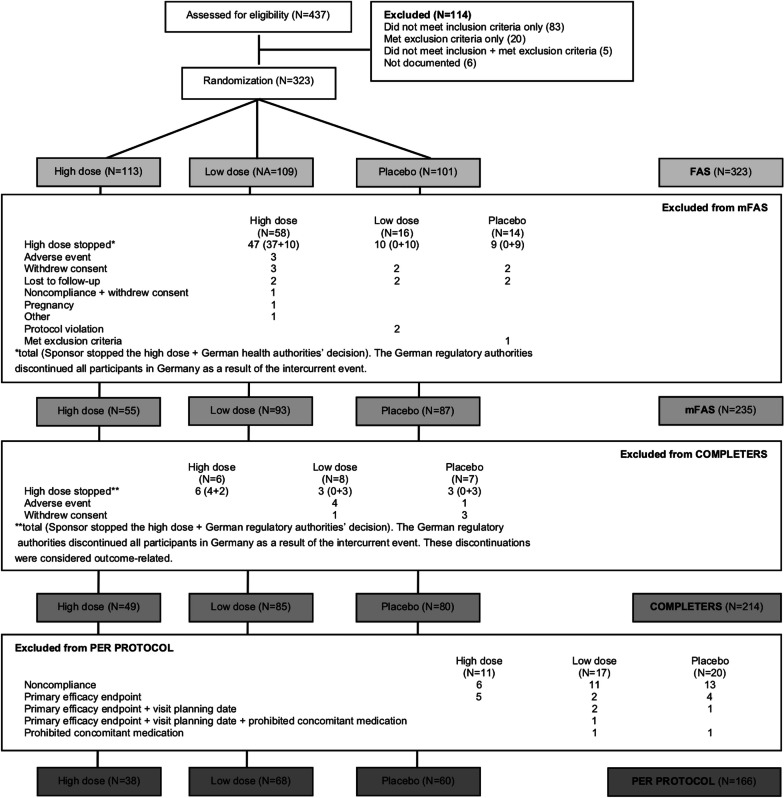
Subject disposition. The full analysis set (FAS) consisted of all randomized subjects. The modified FAS (mFAS) consisted of all randomized subjects except those who dropped out for reasons unrelated to outcome as judged by an independent adjudication committee. The completers population included all subjects who had at least a 12-month visit. The per protocol population consisted of all subjects who had no major protocol violation between randomization and at least the 12-month visit. N is the number of subjects in each group

In total, 437 patients were screened, 323 subjects were randomized, and the modified full analysis set (mFAS) consisted of 235 subjects (Fig. 1). The actual discontinuation rate prior to Visit 5 (month 12) was 33.7%, meaning that the study had a statistical power of 75% rather than 90%, as originally planned. As shown in Table 1 and Additional file 1: Table S1, there were no notable differences between the mFAS and FAS in terms of baseline disease characteristics or demographics. However, the mean treatment duration was slightly reduced in the high-dose arm compared to the low-dose and placebo arms in the mFAS (398.9 ± 59.2 days vs 420.2 ± 86.8 and 418.9 ± 88.7 days, respectively) due to the intercurrent event.

**Table 1:** Subject demographics and baseline characteristics

	High-dose PXT3003^a^		Low-dose PXT3003		Placebo	
	FAS	mFAS	FAS	mFAS	FAS	mFAS
	(N = 113)	(N = 55)	(N = 109)	(N = 93)	(N = 101)	(N = 87)
CMTNS-v2^b^	13.68 (3.19)	13.02 (3.25)	14.13 (3.04)	14.04 (2.99)	13.94 (3.13)	14.01 (3.27)
ONLS score	3.18 (1.11)	3.05 (1.13)	3.39 (1.05)	3.33 (1.05)	3.20 (1.17)	3.23 (1.19)
Age (years)	39.6 (13.9)	41.2 (13.6)	41.0 (12.3)	40.7 (12.4)	42.1 (13.2)	42.3 (13.2)
BMI (kg/m^2^)	25.2 (4.52)	24.9 (4.53)	25.6 (4.72)	25.4 (4.68)	25.4 (4.94)	25.2 (4.90)
*Sex (n, [%])*						
Female	68 (60.2)	34 (61.8)	60 (55.0)	51 (54.8)	62 (61.4)	51 (58.6)
Male	45 (39.8)	21 (38.2)	49 (45.0)	42 (45.2)	39 (38.6)	36 (41.4)
*Race (n, [%])*						
White	110 (97.3)	53 (96.4)	107 (98.2)	91 (97.8)	100 (99.0)	86 (98.9)
Black	1 (0.9)	1 (1.8)	0 (0.0)	0 (0.0)	0 (0.0)	0 (0.0)
Asian	2 (1.8)	1 (1.8)	2 (1.8)	2 (2.2)	1 (1.0)	1 (1.1)

Values other than race and sex are reported as mean (standard deviation)

BMI, body mass index; CMTNS-v2, Charcot–Marie–Tooth Neuropathy Scale version 2; FAS, full analysis set; mFAS, modified full analysis set; ONLS, Overall Neuropathy Limitations Scale

^a^High dose: 6 mg baclofen, 0.70 mg naltrexone, 210 mg D-sorbitol; low dose: 3 mg baclofen, 0.35 mg naltrexone, 105 mg D-sorbitol, administered twice-a-day

^b^CMTNS-v2 total score; in these measurements, N = N − 1 for the high-dose and placebo groups in both the FAS and mFAS

### Efficacy

**Fig. 2:**
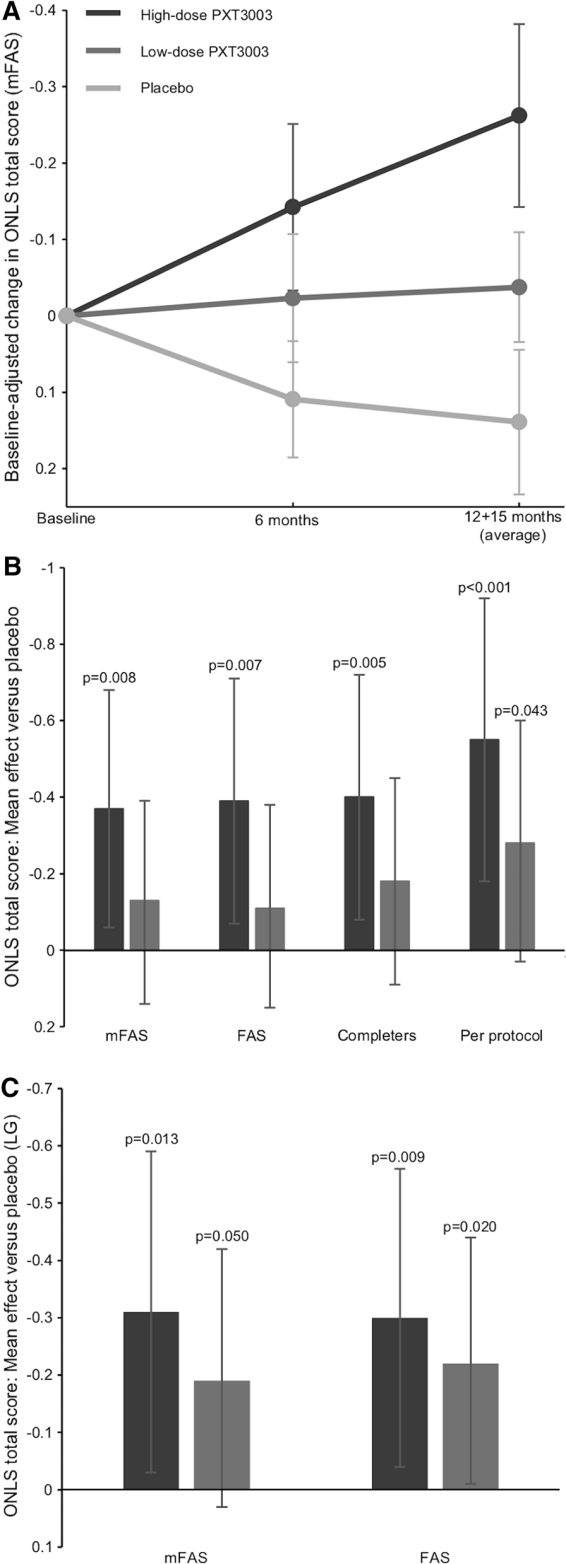
Primary efficacy endpoint results. Mean change in Overall Neuropathy Limitations Scale (ONLS) total score in the modified full analysis set (mFAS, no imputation) high-dose PXT3003 (dark grey), low-dose PXT3003 (medium grey) and placebo (light grey) groups over the course of the study (panel **A**, error bars are standard error of the mean). The treatment effect vs placebo as measured by baseline-adjusted change with multiple imputation in ONLS score is shown in panel **B** for the primary analysis and the first sensitivity analysis (FAS: full analysis set). Panel **C** shows the second sensitivity analysis (LG: longitudinal model). The error bars in **B** and **C** are 97.5% confidence intervals. Axes are reversed to emphasize that lower ONLS scores indicate improvement

From baseline to the end of treatment, the Overall Neuropathy Limitations Scale (ONLS) score decreased (indicating disability improvement) in both the high-dose and low-dose PXT3003 groups but increased (indicating disability worsening) in the placebo group (Fig. 2A). The change in ONLS score was greatest in the high-dose group (Fig. 2A), for which the mean effect was − 0.37 points (*p* = 0.008) (Fig. 2B). The mean effect of the low dose vs placebo was not statistically significant (*p* = 0.287). A responder analysis was performed on the completers population to assess the likelihood of a subject’s total ONLS score not deteriorating. The odds ratio for not deteriorating (improvers and non-decliners combined) was 3.39 (*p* = 0.026, 97.5% CI: [0.99, 11.62]) in the high dose vs placebo and 1.26 (*p* = 0.569, 97.5% CI [0.50, 3.16]) in the low dose vs placebo. Pre-specified analyses also showed a significant dose effect on the relationship between study drug dose and ONLS total score; an effect of − 0.17 points (*p* = 0.013, 95% CI [− 0.31, − 0.04]) was estimated per unit increase in dose.

**Fig. 3:**
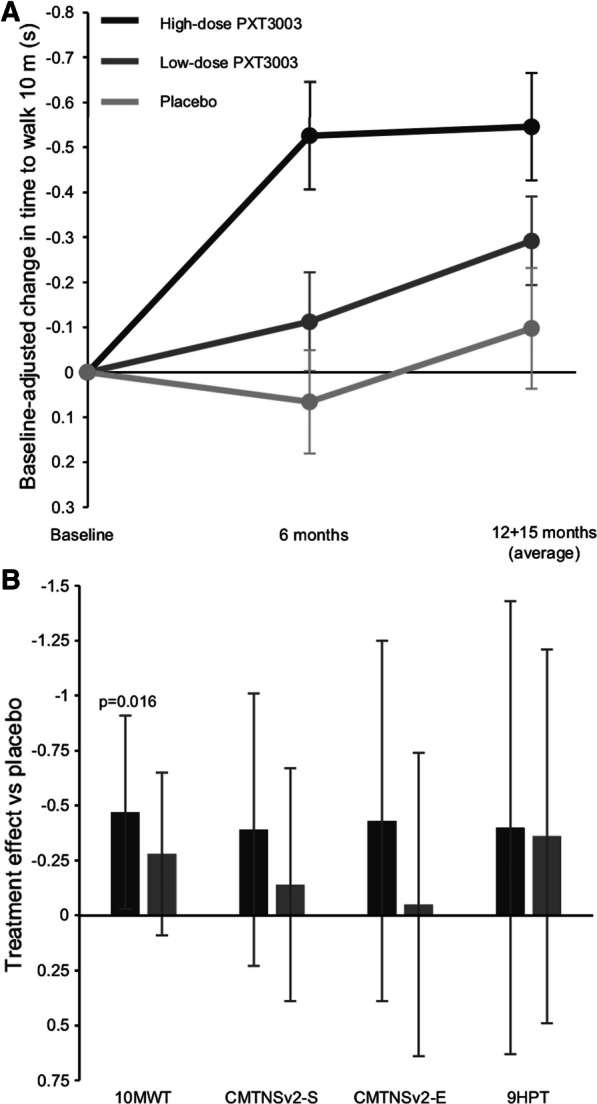
Secondary efficacy endpoints results. Baseline-adjusted mean change in time to walk 10 m in the modified full analysis set (no imputation) for the high-dose, low-dose and placebo groups over the course of the study is shown in panel **A** (error bars are standard errors of the mean). Treatment effect vs placebo as measured by baseline-adjusted change with multiple imputation for all secondary endpoints are shown in panel **B** (error bars are 97.5% confidence intervals). The vertical axis is reversed to show that negative numbers indicate improvement. 10MWT: 10 m walk test (time to walk 10 m), CMTNSv2-S and E: Charcot–Marie–Tooth Neuropathy Scale version 2 Sensory and Examination scores, 9HPT: nine-hole peg test

The point estimate of the treatment effect compared to placebo of all secondary efficacy endpoints was greater for the high-dose group than the low-dose group, although only the treatment effect for the 10-m walk test (10MWT, time to walk 10 m) in the high-dose group (*p* = 0.016) was statistically significant (Fig. 3).

In addition to the results described above, both the high- and low-dose PXT3003 treatment groups showed an increase in the point estimate of the treatment effect compared to placebo but no statistical significance in the majority of exploratory endpoints (Table 2 and Additional file 2: Table S2).

**Table 2:** Treatment effect of high and low-dose PXT3003 vs placebo in exploratory efficacy endpoints (mFAS)

	Test	High-dose PXT3003	Low-dose PXT3003
ONLS	Arm score (↓)	− 0.19 (− 0.43, 0.04)	0.01 (− 0.19, 0.22)
	Leg score (↓)	** − 0.18** (− 0.36, 0.01)	− 0.13 (− 0.29, 0.02)
QMT	Grip strength (↑) (kg)	− 1.49 (− 3.92, 0.93)	0.37 (− 1.64, 2.37)
	Dorsiflexion strength (↑) (Newton)	7.48 (− 3.60, 18.57)	4.09 (− 5.01, 13.20)
Electrophysiology	CMAP (↑) (mV)	− 0.33 (− 1.05, 0.40)	0.14 (− 0.46, 0.74)
	Motor nerve conduction velocity (↑) (m/s)	0.08 (− 1.52, 1.67)	− 0.09 (− 1.42, 1.25)
	Distal motor latency (↓) (ms)	− 0.20 (− 1.58, 1.18)	0.08 (− 1.07, 1.24)
	Radial SNAP (↑) (µV)	− 0.11 (− 1.20, 0.99)	− 0.04 (− 0.96, 0.88)
Quality of life	Mobility (↓)	− 0.07 (− 0.35, 0.20)	− 0.11 (− 0.34, 0.12)
	Self-care (↓)	− 0.12 (− 0.31, 0.06)	** − 0.18** (− 0.34, − 0.03)
	Usual activities (↓)	− 0.02 (− 0.27, 0.23)	− 0.11 (− 0.32, 0.10)
	Pain/discomfort (↓)	0.00 (− 0.25, 0.26)	− 0.12 (− 0.33, 0.09)
	Anxiety/depression (↓)	− 0.04 (− 0.29, 0.22)	− 0.06 (− 0.28, 0.15)
	Visual analog scale (↑) (mm)	2.57 (− 2.36, 7.50)	**4.67** (0.59, 8.75)

Arrows indicate whether a higher (↑) or a lower (↓) value indicates a better clinical condition. Values in parentheses are 97.5% confidence intervals. Bolded values were statistically significant—the change in the ONLS leg score in the high-dose group had *p* = 0.030, the change in the self-care quality of life score in the low-dose group had *p* = 0.007, and the change in the visual analog scale in the low-dose group had *p* = 0.010. No other items were statistically significant. The change from baseline to end of treatment of exploratory endpoints are shown for each treatment group in Additional file 2: Table S2

CMAP, Compound muscle action potential; mFAS, modified full analysis set; SNAP, radial sensory nerve action potential; QMT, quantified muscular testing

### Sensitivity analyses

The treatment effect was consistent between the primary and sensitivity analyses (Fig. 2B, C). The similarity between the mFAS and the FAS in first sensitivity analysis shows that no particular bias was introduced from the use of the mFAS as the primary efficacy population. The per protocol population showed the greatest treatment effect. Notably, the second sensitivity analysis showed the smallest difference between the high-dose and low-dose groups. The second sensitivity analysis also estimated changes of − 0.16 (*p* = 0.006, 95% CI [− 0.27, − 0.05], FAS) and − 0.16 (*p* = 0.008, 95% CI [− 0.28, − 0.04], mFAS) in the ONLS total score per unit increase in drug dose, which was similar to the dose effect estimated in the primary analysis.

### Safety and tolerability

There were no treatment-related serious adverse events. Treatment-emergent adverse events (TEAEs) were reported in a similar proportion of subjects across treatment groups, and most events were mild/moderate in severity (Table 3).

**Table 3:** Summary of treatment-emergent adverse events

	High-dose PXT3003	Low-dose PXT3003	Placebo	*p*
	N = 113	N = 109	N = 101	
Any TEAE	87 (77.0)	89 (81.7)	83 (82.2)	0.581
Treatment-related TEAEs	38 (33.6%)	39 (35.8%)	34 (33.7%)	0.935
Moderate or severe	5 (4.4%)	8 (7.3%)	10 (9.9%)	0.281
TEAE leading to drug withdrawal	6 (5.3%)	6 (5.5%)	6 (5.9%)	1
Treatment-related TEAEs	2 (1.8%)	3 (2.8%)	2 (2.0%)	0.898
Serious TEAEs	3 (2.7%)	10 (9.2%)	5 (5.0%)	0.108
Treatment-related TEAEs	Nil	Nil	Nil	
Leading to drug withdrawal	Nil	1 (0.9%)	Nil	
Deaths	Nil	Nil	Nil	

TEAE, treatment-emergent adverse event

The most frequent TEAEs were related to the gastrointestinal and nervous systems (nausea, diarrhea, headache, and dizziness), with no notable differences between groups (Table 4).

**Table 4:** Adverse events reported in ≥ 2% of subjects in each group^a^

Event^b^	High-dose PXT3003	Low-dose PXT3003	Placebo	*p*^c^
	N = 113	N = 109	N = 101	
Nausea	6 (5.3)	7 (6.4)	3 (3.0)	0.502
Diarrhea	6 (5.3)	3 (2.8)	5 (5.0)	0.620
Headache	6 (5.3)	6 (5.5)	3 (3.0)	0.639
Dry mouth	3 (2.7)	2 (1.8)	4 (4.0)	0.642
Dizziness	3 (2.7)	4 (3.7)	1 (1.0)	0.496
Muscle spasms	3 (2.7)	nil	3 (3.0)	0.223
Nasopharyngitis	3 (2.7)	4 (3.7)	5 (5.0)	0.662
Dyspepsia	2 (1.8)	1 (0.9)	2 (2.0)	0.868
Fatigue	1 (0.9)	6 (5.5)	4 (4.0)	0.132
Somnolence	1 (0.9)	5 (4.6)	1 (1.0)	0.185
Constipation	1 (0.9)	3 (2.8)	1 (1.0)	0.536
Arthralgia	1 (0.9)	3 (2.8)	2 (2.0)	0.608
Asthenia	1 (0.9)	1 (0.9)	2 (2.0)	0.693
Tinnitus	1 (0.9)	1 (0.9)	2 (2.0)	0.693
Abdominal pain	1 (0.9)	Nil	2 (2.0)	0.311
Weight increased	Nil	Nil	3 (3.0)	0.030

^a^Data reported as number of subjects (%)

^b^Coded according to preferred terms in the Medical Dictionary for Regulatory Activities, version 18.1 or later

^c^Values calculated using Fisher’s exact test. Please note that there is no correction for multiple testing, thus no control of type I error rate

No adverse events were reported in relation to the appearance of crystals in the high-dose formulation. There were no notable changes during the study in laboratory parameters and no notable differences were observed between the treatment groups. Treatment compliance was 90.5%, 94.4% and 91.7% in the high-dose, low-dose and placebo groups, respectively, which indicates good tolerability.

## Discussion

### Outcomes

Despite the intercurrent event that reduced the statistical power of the study, the high-dose PXT3003 group demonstrated statistically significant improvements in subjects’ ONLS scores (primary endpoint) and 10MWT compared to placebo. The treatment effect of the low-dose group was not statistically significant. The ONLS is an ordinal scale; an increase in the leg score from 2 to 3 means that a patient goes from walking 10 m independently but with an abnormal gait to requiring unilateral support, and an increase from 4 to 5 means a patient goes from needing bilateral support to a wheelchair to move 10 m [19]. Because the ONLS is based on functional motor tasks, stabilization of the ONLS score would be expected to be beneficial in a chronic progressive disease. Therefore, even though the choice of a 0.3-point change in ONLS score was pre-specified as the anticipated treatment effect based on observations from the phase 2 study [18] and for statistical reasons rather than established clinical evidence, the statistically significant and measurable change in ONLS scores between the high-dose PXT3003 and placebo groups is promising. Considering the statistical significance difference in favor of the high-dose group, the good safety profile, and the dose–effect relationship on ONLS score, future studies may focus only on the high-dose formulation.

The efficacy demonstrated in the primary analysis was supported by the longitudinal model used in the second sensitivity analysis. The second sensitivity analysis showed the smallest difference between the high-dose and low-dose groups and estimated a change of − 0.31 points per year (*p* = 0.013) in the high-dose group, which suggests that PXT3003 may have a promising long-term treatment effect. Following the natural progression of CMT1A, patient condition is expected to deteriorate over time—a previous study in 72 untreated CMT1A patients followed for up to eight years estimated an annual increase in the CMTNS total score of 0.686 points [20]. While the results of this study and the second sensitivity analysis in particular are promising in terms of treatment effect, long-term data are needed to assess the safety and efficacy of PXT3003 on timescales comparable with the slow disease progression. Such data is being obtained from an open-label continuation study (ClinicalTrials.gov identifier: NCT03023540).

The secondary endpoints all showed improvement in the point estimates in the PXT3003 groups compared to placebo, but the improvement was only statistically significant in the 10MWT. In general, a lack of statistical significance could arise from either a lack of effect or from an inability to detect an effect in an underpowered study. Given that this study suffered from an unexpectedly high discontinuation rate, the latter probably strongly influenced the estimates of statistical significance (*p*-values and confidence intervals). In fact, the detection of any statistically significant change in the secondary endpoints was unexpected. However, because CMT1A tends to affect legs and feet more and earlier than hands and arms, the fact that the statistically significant secondary endpoint was the 10MWT was not surprising. The improvement in the 10MWT was consistent with improvement in the ONLS leg score (exploratory endpoint) and suggests that subjects taking high-dose PXT3003 experienced improved mobility as a result of the treatment.

Both PXT3003 doses demonstrated an acceptable safety profile. This is consistent with previous safety evaluations [18]; side effects are likely to be reduced in combination therapies of low-dose constituent drugs [21]. All adverse events were reported at comparable rates in all treatment groups and no subject reported any objective PXT3003-related adverse event. Considering the higher efficacy and similar safety profile of high-dose PXT3003 compared to low-dose PXT3003, high-dose PXT3003 has a better risk/benefit ratio than low-dose PXT3003. The safety profile of the high dose will be better understood when the data from the ongoing open-label continuation study becomes available later in 2021, but to date there have been no new safety signals or concerns in patients taking high-dose PXT3003.

The open-label continuation study comprises 187 subjects and measures the incidence of TEAEs as the primary endpoint and includes efficacy measurements as secondary endpoints. Efficacy measurements are the same as those reported here, and include the ONLS, the 10MWT, the nine-hole peg test, CMTNS-v2, quantified muscular testing, compound muscle action potential, sensory nerve action potential, nerve conduction velocity, and quality-of-life scales. All subjects who completed the phase 3 study reported here were invited to participate and after the completion of the phase 3 study, so were all subjects who were discontinued from the high-dose group. At the end of the continuation study, subjects will have been treated for up to 48 months, which should help elucidate the long-term risk/benefit profile and complement the data obtained from this phase 3 study.

### Drawbacks and limitations

The intercurrent event, which led to a smaller sample size, caused the statistical power of the study to drop from the expected 90% to 75% for the primary endpoint. It is noteworthy that the high-dose group, which suffered from the most subject discontinuations, was also the group that showed the greatest treatment effect. Two effects could have caused the high-dose treatment effect to be impacted: potential partial unblinding of the placebo group due to the discontinuation of the high-dose group and potential bias from the use of the mFAS. Although the crystallization was first reported by some subjects, the risk of unblinding was limited by the fact that a) no subject was aware of the identity of the affected formulation (high-dose PXT3003, low-dose PXT3003, or placebo) at the time of the disclosure of the crystals, b) subjects being discontinued due to the intercurrent event were not informed of their group assignment even when they were discontinued, and c) due to the decision made by German regulatory authorities, subjects were discontinued also from the low-dose and placebo group. Furthermore, considering that a significant number of subjects in all groups had already completed the study at the time of the intercurrent event, unblinding could only have affected a subset of subjects in the low-dose and placebo groups. Those remaining subjects would have acquired no further knowledge as to their allocation to the placebo or low-dose group, which makes unblinding unlikely to have strongly impacted the estimated high-dose treatment effect. With regards to any potential bias arising from the use of the mFAS, the mFAS was defined to reduce the need for imputation and still adhere to the intention-to-treat principle. A sensitivity analysis was included to check any bias arising from the use of an alternate analysis population, and the results suggest that little or no bias was introduced by the use of the mFAS. Therefore, it is unlikely that the significant treatment effect observed for the high dose was artificially inflated by unblinding or bias from the use of the mFAS.

Also due to the reduced sample size, the study had limited power to detect statistically significant changes in many of the secondary and exploratory endpoints. This drop in statistical power could explain the lack of statistical significance in many of the secondary and exploratory endpoints as opposed to the null hypotheses being true. Three of 20 exploratory endpoints demonstrated statistically significant changes; although these could be false-positives, it is statistically unlikely that all three would be significant by chance alone. It is therefore likely that at least one of these truly reflect a measurable treatment effect. Moreover, the unexpectedly small sample size could be masking other statistically significant changes. None of the three statistically significant exploratory endpoints were physiological measurements, and this could be either because the physiological parameters were not affected by the treatment or because the treatment effect was not detectable given the small sample size.

The formation of crystals was limited to the high-dose formulation. The crystals formed as a result of precipitation of some of the active ingredients, which did not result in any degradation products but did lead to a slight decrease in potency. Because the low-dose formulation did not present with stability issues, future study subjects could be given the high-dose treatment in the form of a double volume of the low-dose formulation; i.e., 10 mL low-dose PXT3003 instead of 5 mL high-dose PXT3003, orally twice daily. Such a dosage, which should completely resolve the issue, is being used in the open-label continuation study and will be investigated in a confirmatory phase 3 clinical trial.

## Conclusions

Subjects receiving PXT3003 demonstrated stabilization of disease progression by showing improvement or no change in ONLS scores, and this positive result is further validated by the improvement observed in the 10MWT. It is important to note that the treatment benefit observed over the limited duration of a clinical trial should be evaluated within the context of the known and well-characterized progression of CMT1A. In other words, if PXT3003 delivers a sustained therapeutic effect over a longer treatment period and achieves disease stabilization by modifying its chronically progressive course, PXT3003 may address a current unmet need in the treatment of CMT1A. A confirmatory study investigating high-dose PXT3003 as a double-volume of the low-dose PXT3003 formulation was anticipated to be initiated in the first half of 2021.

## Methods

This phase 3 study (ClinicalTrials.gov identifier: NCT02579759, December 2015–March 2018) was conducted in accordance with the Declaration of Helsinki and Good Clinical Practice guidelines. A total of thirty centers in Belgium, Canada, France, Germany, the Netherlands, Spain, the United Kingdom and the United States were involved. Ethical approval was obtained from institutional review boards or ethics committees. All subjects provided written informed consent.

### Overview

Eligible subjects were men or women aged 16–65 years, with a genetically proven diagnosis of CMT1A of mild to moderate severity (i.e., a CMT Neuropathy Score version 2 [CMTNS-v2] score of 2–18), clinically confirmed muscle weakness in at least the foot dorsiflexors, and motor nerve conduction of at least 15 m/s in the ulnar nerve. Subjects diagnosed with any other type of concomitant peripheral neuropathy, such as diabetic neuropathy, another significant neurological disease or major systemic disease were excluded. Full eligibility criteria are provided in Additional file 3: Table S3.

After a screening period of up to four weeks, eligible subjects were randomly allocated in a 1:1:1 ratio to receive high-dose PXT3003, low-dose PXT3003 or placebo. Randomization was done using a block scheme stratified by study center, and study treatments were numbered according to a material randomization list, which was separate from the subject randomization list. The randomization code, which was not available to the study centers, the Sponsor or the subjects, was created by an independent statistician who was not involved in any other activity related to the study. All treatments were liquid formulations indistinguishable by look or taste and packaged in identical kits.

After randomization, subjects entered the treatment phase. To improve tolerability, subjects began by taking a half dose (2.5 mL) orally twice daily for two weeks and then escalating to 5 mL (full dose) orally twice daily for the remainder of the study. The treatment period lasted for up to 15 months. To avoid excluding subjects who completed a significant part of the treatment period, a functional measurement at the 12-month visit was sufficient for a subject to have an evaluable endpoint. The mean of measurements at the 12- and 15-month visits was used as the endpoint for subjects who completed the full 15 months of treatment.

**Fig. 4:**
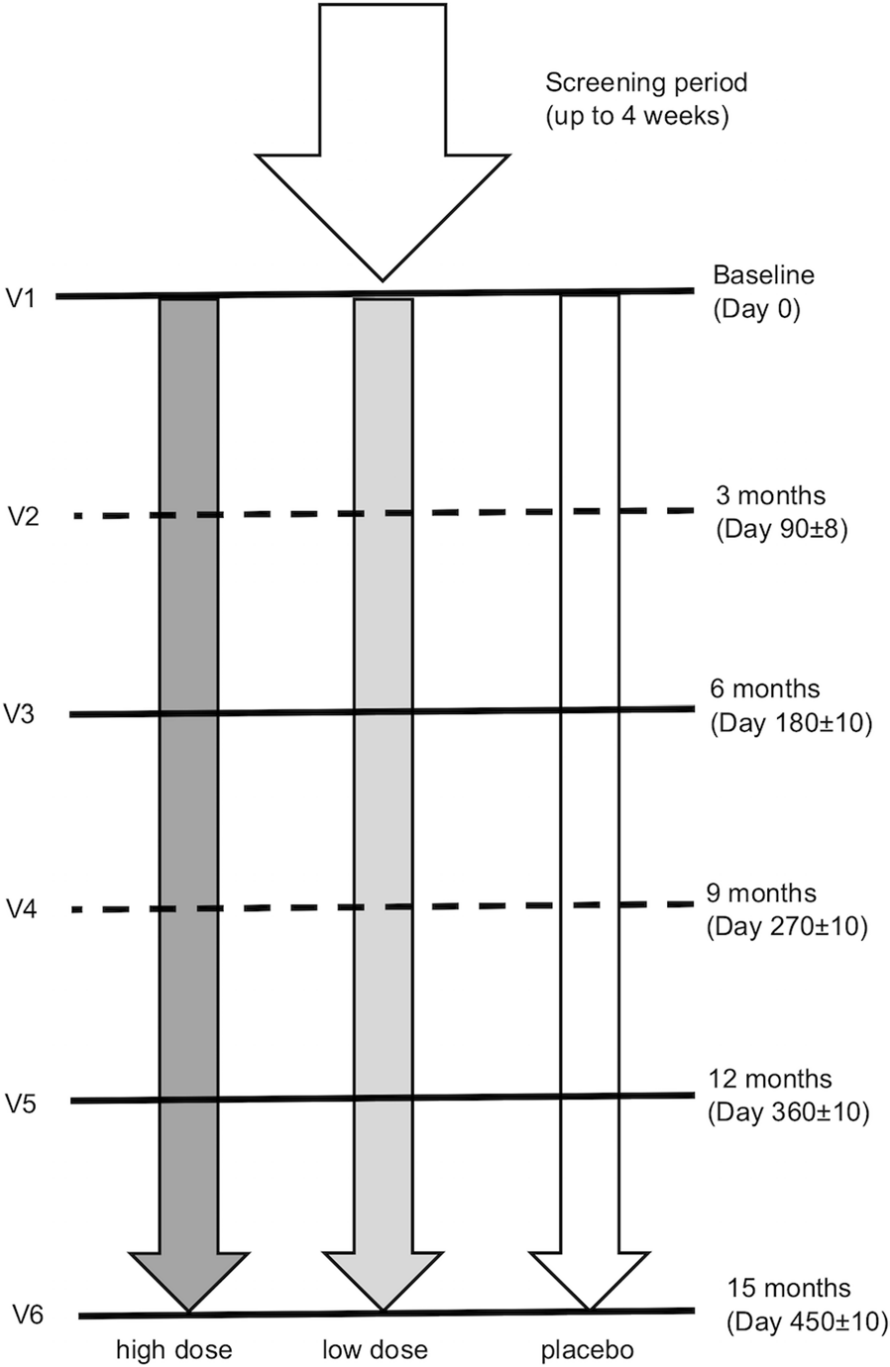
Study design. Subjects were randomized to one of three treatment arms after a screening period of maximum four weeks. During the treatment period, subjects attended six visits, two of which (dashed line) could be done by telephone

During the study, subjects were asked to attend up to six visits (V1 to V6) (Fig. 4) after the initial screening visit (V0), during which informed consent was obtained and demographic data, medical history as well as baseline disease characteristics were recorded. Eligibility criteria were evaluated at both V0 and at the randomization visit (V1). Physical and neurological examinations, nerve conduction studies, vital signs (blood pressure and heart rate) measurements and CMTNS-v2 evaluations were performed at V0, V1, V3, V5 and V6. In V1, V3, V5 and V6, subjects were assessed using the ONLS, nine-hole peg test, 10MWT, quantified muscle testing (dorsiflexion and grip strength), and the EQ-5D-5L quality of life questionnaire, which included a visual analog scale ranging from “the best health you can imagine” to “the worst health you can imagine.” Safety was assessed at each treatment visit (V1–V6), including physical and neurological examinations, electrocardiography, clinical laboratory tests, vital signs measurements and recording of adverse events.

### Objectives and outcomes

The primary objective of this study was to evaluate the efficacy of the two PXT3003 doses compared to placebo. The primary efficacy endpoint was the treatment effect on disability improvement as measured by the change in the ONLS total score from baseline the end of treatment. The ONLS was selected as the primary endpoint given that regulatory authorities require the use of clinical outcome measures for the primary endpoint. Furthermore, the 136th European Neuromuscular Committee meeting in 2006 [22] recommended the ONLS as the core disability scale and as a secondary endpoint, even though it discouraged the use of disability scales as primary endpoint measures. The ONLS, which ranges from 0 (no disability) to 12 (maximum disability), was originally proposed as an improved form of the ODSS [19] and is considered a reliable disability scale for patients with CMT with acceptable inter- and intra-rater reliability [22–24]. Secondary efficacy endpoints included functional tests (10MWT and nine-hole peg test) as well as the CMTNS-v2 Sensory score (sum of items 1, 4 and 5: sensory symptoms, pinprick sensibility and vibration) and Examination score (sum of items 1–7: sensory symptoms, motor symptoms [legs], motor symptoms [arms], pinprick sensibility, vibration, strength [legs] and strength [arms]). Exploratory efficacy endpoints included ONLS arm and leg subitems, the odds of a subject’s ONLS score improving or remaining stable in response to treatment, quantified muscular testing, electrophysiological parameters and quality of life measures. These physiological parameters were chosen as exploratory endpoints as they have been shown to change as CMT1A progresses [25].

The secondary objective was to evaluate the safety and tolerability of the two PXT3003 doses compared to placebo. Safety and tolerability were assessed by the incidence and severity of TEAEs, their relationship to the study drug, the incidence of TEAEs that led to withdrawal of study drug, the incidence of serious adverse events and changes in physical and neurological examinations, vital signs, 12-lead electrocardiograms and laboratory data. Treatment compliance was assessed by counting the number of bottles dispensed and the number of used bottles returned.

### Statistical methods

All statistical analyses, performed by DICE NV using Statistical Analysis System (SAS) version 9.4 or later, were conducted in accordance with the statistical analysis plan, which was written before database lock and study unblinding. Treatment group allocation data were stored in a dedicated file sent to the analysts after database lock.

Considering the slow progression of CMT1A and the limited duration of a clinical trial, a 0.3-point mean change in ONLS score over the course of the study was pre-specified as clinically meaningful based on the results from the phase 2 study [18] and a Cohen’s d-value calculation [26, 27]. A sample size of 89 subjects per group should make such a difference detectable versus placebo with a power of 90%. Because a monotonic dose effect was not assumed, each dose was compared at a 2.5% significance level to preserve an overall false–positive of 5%. The dropout rate was anticipated to be 10% [18, 28] meaning that 100 subjects needed to be recruited to each treatment arm and at least 300 subjects needed to be recruited in total.

The primary efficacy analysis was performed using a linear mixed model analysis of covariance on the summary mean of ONLS scores at 12 and 15 months adjusted for baseline ONLS scores. Baseline ONLS scores and treatment were the fixed factors and center was admitted as a random effect. Missing data was to be imputed using a multiple imputation method based on the placebo group distribution. Secondary and exploratory endpoints were analyzed analogously to the primary endpoint.

Two sensitivity analyses were also planned. In the first sensitivity analysis, the primary analysis was performed on the full analysis set, the completers and per protocol populations (Fig. 1) to check for any significant variation in treatment effect by population. The second sensitivity analysis used a longitudinal model without imputation instead of an analysis of covariance in determining efficacy. The longitudinal model hypothesized a linear time–effect relationship and used the actual time of visits to estimate differences in the slope of ONLS evolution in the randomized treatment arms. A sensitivity analysis (described in Additional file 4: Table S4) investigating the impact of subjects’ age and sex on the treatment effect was also conducted.

During the study, the high-dose PXT3003 formulation was found to have a stability issue as crystals formed inside some bottles. As a result of this intercurrent event, all subjects in Germany who had not already completed the study were discontinued (n = 62), as required by the German regulatory authorities, as were all ongoing subjects in the high-dose PXT3003 group (n = 59) in all other countries, following the sponsor’s decision. Although the crystallization did not cause additional safety concerns, it had a significant impact on the subject discontinuation rate. Because a large proportion of subjects discontinued the study due to the intercurrent event rather than for reasons related to outcome, an mFAS was defined before database lock. Defining an mFAS was in line with the intent-to-treat approach as it included all subjects in the full analysis set (FAS, i.e., all randomized subjects) except for those who discontinued the study for reasons not related to clinical outcome (e.g., adverse event). An adjudication committee, consisting of two independent clinical neurologists blinded to both the treatment and the cause leading to discontinuation, assessed each discontinuation and classified them as outcome-related or outcome-unrelated. If consensus was not reached within the committee, the discontinuation was assumed outcome-related and included in the mFAS. The mFAS included 25/62 subjects discontinued due to the German regulatory authorities’ decision and 18/59 subjects discontinued following the sponsor’s decision. Outcome-related missing data were imputed as anticipated using a linear model with a multiple imputation method based on the placebo group distribution, while outcome-unrelated missing data in the FAS was imputed based on the distribution in the subject’s treatment arm.

The sensitivity analyses defined in the study protocol were modified before database lock and unblinding to account for the use of the mFAS as the primary analysis population. The FAS was added as a population to the first sensitivity analysis to investigate the bias arising from the use of the mFAS as the primary analysis population. The second sensitivity analysis, which was expected to correct for the variation by group in the treatment duration that the unexpectedly high discontinuation rate caused, was performed on both the mFAS and FAS. Because the longitudinal model used more data points than the analysis of covariance, and time–effect relationship data from the prior phase 2 study appeared to be linear, it was also expected to have greater statistical power.

The original analysis plan foresaw the use of the Dunnett procedure to correct for multiple testing. However, as a result of the intercurrent event and difficulties in using this procedure with multiple imputation in the longitudinal model, a Bonferroni correction based on unadjusted *p*-values and 97.5% confidence intervals was used instead. As Bonferroni corrections are more conservative than the Dunnett procedure, this change did not increase the type I error rate [29, 30].

Safety was analyzed using descriptive statistics and Fisher’s exact test without correction for multiple testing (and therefore no control of type I error rate). All randomized subjects who had received at least one dose of the study drug were included in the analysis population.

### Supplementary Information

**Additional file 1**. Demographic bias analysis.

**Additional file 2**. Exploratory efficacy endpoints (change from baseline to end of treatment).

**Additional file 3**. Full eligibility criteria.

**Additional file 4**. Sensibility analysis: impact of age and sex on treatment effect.

## Data Availability

The datasets used and/or analyzed during the current study are available from the corresponding author on reasonable request.

## Abbreviations

10MWT: 10-M walk test (time to walk 10 m)
CMT1A: Charcot—Marie—Tooth disease type 1A
FAS: Full analysis set
mFAS: Modified full analysis set
ONLS: Overall Neuropathy Limitations Scale
TEAEs: Treatment-emergent adverse events
