# Correction to: A double-blind, placebo-controlled, randomized trial of PXT3003 for the treatment of Charcot–Marie–Tooth type 1A

**DOI:** 10.1186/s13023-024-03110-3

**Published:** 2024-04-01

**Authors:** Shahram Attarian, Peter Young, Thomas H. Brannagan, David Adams, Philip Van Damme, Florian P. Thomas, Carlos Casanovas, Jafar Kafaie, Céline Tard, Maggie C. Walter, Yann Péréon, David Walk, Amro Stino, Marianne de Visser, Camiel Verhamme, Anthony Amato, Gregory Carter, Laurent Magy, Jeffrey M. Statland, Kevin Felice

**Affiliations:** 1 Reference Center for Neuromuscular Disorders and ALS, CHU La Timone, Marseille, France; 2 Department of Neurology, Medical Park Bad Feilnbach, Bad Feilnbach, Germany; 3 Columbia University Medical Center, The Neurological Institute, New York, USA; 4 French Reference Center for Rare Peripheral Neuropathies, Service de Neurologie Adulte, APHP, CHU Bicêtre, Le Kremlin Bicêtre, France; 5 Department of Neurology, University Hospitals Leuven, KU, Leuven, Belgium; 6 Center for Brain & Disease Research, VIB, Leuven, Belgium; 7 Department of Neurology, Hackensack University Medical Center, Hackensack, USA; 8 Department of Neurology, Saint Louis University School of Medicine, St. Louis, USA; 9 Neuromuscular Unit, Neurology Department, Bellvitge University Hospital, Barcelona, Spain; 10 Neurometabolic Diseases Group, Bellvitge Research Institute (IDIBELL) and CIBERER, Barcelona, Spain; 11 U1171, Centre de référence des maladies neuromusculaires Nord Est Ile de France, Hôpital Salengro CHU de Lille, Lille, France; 12 Department of Neurology, Friedrich-Baur-Institute, Ludwig-Maximilians-University of Munich, Munich, Germany; 13 Centre de Référence Maladies Neuromusculaires AOC, Filnemus, Euro-NMD, CHU Nantes, Hôtel-Dieu, Nantes, France; 14 Clinical Neuroscience Research Unit, University of Minnesota, Minneapolis, USA; 15 University of Michigan Health System, Ann Arbor, MI USA; 16 Department of Neurology, Amsterdam University Medical Centres, University of Amsterdam, Amsterdam Neuroscience, Amsterdam, The Netherlands; 17 Department of Neurology, Brigham and Women’s Hospital, Boston, USA; 18 St. Luke’s Rehabilitation Institute, Physical Medicine and Rehabilitation, Spokane, USA; 19 CHU Dupuytren, Limoges, France; 20 University of Kansas Medical Center, Kansas City, USA; 21 Department of Neuromuscular Medicine, Hospital for Special Care, New Britain, USA

**Correction to: Orphanet Journal of Rare Diseases (2021) 16:433**

10.1186/s13023-021-02040-8

Following publication of the original article [1], we have been notified that we should add one more author to the authors’ group:

Jafar Kafaie 8

8 Department of Neurology, Saint Louis University School of Medicine, St. Louis, USA

The original article was updated.
