# Supplementary material for: A double-blind, placebo-controlled, randomized trial of PXT3003 for the treatment of Charcot–Marie–Tooth type 1A
Source: Orphanet J Rare Dis. 2021 Oct 16;16:433. doi: 10.1186/s13023-021-02040-8 (PMC8520617; doi:10.1186/s13023-021-02040-8)
Supplement: Supplementary file 1 — Additional file 1. Demographic bias analysis. [file 13023_2021_2040_MOESM1_ESM.docx]

A double-blind, placebo-controlled, randomized trial of PXT3003 for the treatment of Charcot–Marie–Tooth type 1A

**Additional file 1**

Whether any bias arose from differences in baseline characteristics between the groups was tested using ANOVA for continuous variables (e.g., age) and Fisher’s exact test for categorical variables (e.g., gender). The p-values of the tests, reported in Table S1, suggest that there were no statistically significant differences between the groups at baseline.

Table S1. Demographic bias analysis

|  | **FAS** | | | | **mFAS** | | |  |
| --- | --- | --- | --- | --- | --- | --- | --- | --- |
|  | High-dose PXT3003^a^ | Low-dose PXT3003 | Placebo | p^c^ | High-dose PXT3003^a^ | Low-dose PXT3003 | Placebo | p^c^ |
|  | (N=113) | (N=109) | (N=101) |  | (N=55) | (N=93) | (N=87) |  |
| CMTNS-v2^b^ | 13.68 (3.19) | 14.13 (3.04) | 13.94 (3.13) | 0.561 | 13.02 (3.25) | 14.04 (2.99) | 14.01 (3.27) | 0.122 |
| ONLS score | 3.18 (1.11) | 3.39 (1.05) | 3.20 (1.17) | 0.284 | 3.05 (1.13) | 3.33 (1.05) | 3.23 (1.19) | 0.344 |
| Age (years) | 39.6 (13.9) | 41.0 (12.3) | 42.1 (13.2) | 0.384 | 41.2 (13.6) | 40.7 (12.4) | 42.3 (13.2) | 0.72 |
| BMI (kg/m^2^) | 25.2 (4.52) | 25.6 (4.72) | 25.4 (4.94) | 0.794 | 24.9 (4.53) | 25.4 (4.68) | 25.2 (4.90) | 0.872 |
| Sex (n, [%]) |  |  |  | 0.609 |  |  |  | 0.71 |
| Female | 68 (60.2) | 60 (55.0) | 62 (61.4) |  | 34 (61.8) | 51 (54.8) | 51 (58.6) |  |
| Male | 45 (39.8) | 49 (45.0) | 39 (38.6) |  | 21 (38.2) | 42 (45.2) | 36 (41.4) |  |
| Race (n, [%]) |  |  |  | NA^d^ |  |  |  | NA^d^ |
| White | 110 (97.3) | 107 (98.2) | 100 (99.0) |  | 53 (96.4) | 91 (97.8) | 86 (98.9) |  |
| Black | 1 (0.9) | 0 (0.0) | 0 (0.0) |  | 1 (1.8) | 0 (0.0) | 0 (0.0) |  |
| Asian | 2 (1.8) | 2 (1.8) | 2 (1.8) |  | 1 (1.8) | 1 (1.8) | 1 (1.8) |  |

Values other than race and sex are reported as mean (standard deviation).

^a^ High dose: 6 mg baclofen, 0.70 mg naltrexone, 210 mg D-sorbitol; low dose: 3 mg baclofen, 0.35 mg naltrexone, 105 mg D-sorbitol, administered twice daily.

^b^ CMTNS-v2 total score; in these measurements, N=N−1 for the high-dose and placebo groups in both the FAS and mFAS.

^c^ *p-*value is given by an ANOVA for continuous variables and by a Fisher exact test for categorical variables.

^d^ NA: not applicable given the number of subjects in groups other than “White”.
