# Supplementary material for: A double-blind, placebo-controlled, randomized trial of PXT3003 for the treatment of Charcot–Marie–Tooth type 1A
Source: Orphanet J Rare Dis. 2021 Oct 16;16:433. doi: 10.1186/s13023-021-02040-8 (PMC8520617; doi:10.1186/s13023-021-02040-8)
Supplement: Supplementary file 2 — Additional file 2. Exploratory efficacy endpoints (change from baseline to end of treatment). [file 13023_2021_2040_MOESM2_ESM.docx]

A double-blind, placebo-controlled, randomized trial of PXT3003 for the treatment of Charcot–Marie–Tooth type 1A

**Additional file 2**

Table S2. Exploratory efficacy endpoints: Change from baseline to end of treatment.

|  | **Test** | **High-dose PXT3003** | **Low-dose PXT3003** | **Placebo** |
| --- | --- | --- | --- | --- |
| ONLS | Arm score (↓) | −0.13±0.64 | −0.01±0.56 | 0.01±0.71 |
|  | Leg score (↓) | −0.07±0.43 | −0.05±0.49 | 0.14±0.59 |
| QMT | Grip strength (↑) (kg) | −1.46±8.11 | 0.34±6.37 | 0.20±7.77 |
|  | Dorsiflexion strength (↑) (Newton) | 9.72±32.75 | −2.35±36.45 | −3.93±31.87 |
| Electrophysiology | CMAP (↑) (mV) | −0.03±1.25 | 0.41±2.08 | 0.20±1.83 |
|  | Motor nerve conduction velocity (↑) (m/s) | 0.23±4.34 | 0.07±2.97 | −0.07±4.82 |
|  | Distal motor latency (↓) (ms) | −1.35±9.37 | −0.45±8.40 | −3.25±14.13 |
|  | Radial SNAP (↑) (µV) | 0.05±2.18 | 0.38±3.26 | 0.47±2.39 |
| Quality of life | Mobility (↓) | −0.04±0.94 | −0.14±0.67 | −0.01±0.59 |
|  | Self-care (↓) | 0.07±0.40 | −0.07±0.48 | 0.12±0.45 |
|  | Usual activities (↓) | 0.02±0.60 | −0.20±0.69 | −0.01±0.69 |
|  | Pain/discomfort (↓) | −0.01±0.61 | −0.24±0.79 | −0.06±0.62 |
|  | Anxiety/depression (↓) | 0.00±0.86 | −0.03±0.52 | 0.01±0.71 |
|  | Visual analog scale (↑) (mm) | 0.41±18.39 | 3.46±15.01 | −1.72±11.54 |

Arrows indicate whether a higher (↑) or a lower (↓) value indicates a better clinical condition. Values are presented as mean±standard deviation. CMAP: Compound muscle action potential; mFAS: modified full analysis set; SNAP: radial sensory nerve action potential; QMT: quantified muscular testing
