# Supplementary material for: A double-blind, placebo-controlled, randomized trial of PXT3003 for the treatment of Charcot–Marie–Tooth type 1A
Source: Orphanet J Rare Dis. 2021 Oct 16;16:433. doi: 10.1186/s13023-021-02040-8 (PMC8520617; doi:10.1186/s13023-021-02040-8)
Supplement: Supplementary file 3 — Additional file 3. Full eligibility criteria. [file 13023_2021_2040_MOESM3_ESM.docx]

A double-blind, placebo-controlled, randomized trial of PXT3003 for the treatment of Charcot–Marie–Tooth type 1A

**Additional file 3**

Table S3. Full eligibility criteria

| **Inclusion criteria:** | |
| --- | --- |
| 1. | Male or female aged 16–65 years |
| 2. | Genetically proven diagnosis of CMT1A |
| 3. | Mild to moderate severity assessed by the CMTNS-v2 (score <2 and ≥18) |
| 4. | Muscle weakness in at least foot dorsiflexion (clinical assessment) |
| 5. | Motor nerve conduction of the ulnar nerve >15 m/s |
| 6. | A written informed consent to participate in the study was signed by the subject who was willing and able to comply with all the study procedures and the scheduled visits |
| **Exclusion criteria:** | |
| 1. | Presenting with any other associated cause of peripheral neuropathy (e.g., diabetes) |
| 2. | Presenting with another significant neurological disease or a concomitant major systemic disease |
| 3. | Having a clinically significant history of an unstable medical illness in the last 30 days (e.g., unstable angina or cancer) that might jeopardize study participation |
| 4. | Having significant hematologic disease, hepatitis, liver failure or renal failure |
| 5. | Undergone limb surgery within six months before randomization or planned limb surgery before study completion |
| 6. | Having clinically significant abnormalities at the pre-study clinical examination, routine evaluations and electrocardiogram |
| 7. | Having elevated aspartate aminotransferase/alanine amino transferase (more than 3 times the upper limit of normal) and/or elevated serum creatinine levels (more than 1.25 times the upper limit of normal) |
| 8. | Having a history of recent alcohol or drug abuse or non-adherence with any treatment or other experimental protocols |
| 9. | Using unauthorized concomitant treatments including (but not limited to) baclofen, naltrexone, pharmaceutical D-sorbitol, opioids, levothyroxine, and potentially neurotoxic drugs (e.g., amiodarone, chloroquine, cancer drugs susceptible to induce a peripheral neuropathy), but participants who could and agreed to stop these medications four weeks before randomization and for the duration of the study could be included |
| 10. | Women of child-bearing potential who were pregnant, breastfeeding, not using highly effective method(s) of birth control (i.e., a failure rate of less than 1% per year) throughout the study and/or unwilling to be tested for pregnancy |
| 11. | History of safety issues or known hypersensitivity to baclofen, naltrexone, or D-sorbitol, or to any of the individual components of PXT3003 |
| 12. | Having any contraindication to the use of baclofen (e.g. porphyria), naltrexone or D-sorbitol, as defined in the national product labels |
| 13. | Suspected inability to complete the study follow up (e.g. being a transient visitor, tourist or any participant for whom the follow-up evaluation would have been too difficult to appreciate for any reason) |
| 14. | Having limited mental capacity or psychiatric disease that rendered the participant unable to provide written informed consent or comply with evaluation procedures |
| 15. | Having participated in another investigational drug(s) trial within the past 30 days |
| 16. | Being of the same family and living in the same household as a participant already included in the study as such could result in unblinding that could have jeopardized the interpretation of the study results |
