# Supplementary material for: A double-blind, placebo-controlled, randomized trial of PXT3003 for the treatment of Charcot–Marie–Tooth type 1A
Source: Orphanet J Rare Dis. 2021 Oct 16;16:433. doi: 10.1186/s13023-021-02040-8 (PMC8520617; doi:10.1186/s13023-021-02040-8)
Supplement: Supplementary file 4 — Additional file 4. Sensibility analysis: impact of age and sex on treatment effect. [file 13023_2021_2040_MOESM4_ESM.docx]

A double-blind, placebo-controlled, randomized trial of PXT3003 for the treatment of Charcot–Marie–Tooth type 1A

**Additional file 4**

**Sensitivity analysis: the effect of covariables on treatment effect**

Investigating covariable effects, such as age and sex, is often useful to better understand the treatment effect. Therefore, a sensibility analysis of the primary analysis, a baseline-adjusted analysis of covariance, taking these covariables into account was pre-specified in the statistical analysis plan. As shown in Table S4, the results of this covariable analysis suggest that no covariable impacted the treatment effect, *i.e.,* the treatment effect was the same regardless of a subject’s age or sex. This was also true for the placebo group (data not shown). When adjusting for age or sex, the effect size of the high dose remained exactly the same (same estimate, same p-value; compare with Fig 2B), and, as shown in the second part of the table, the effects of the covariables were not statistically relevant. Because the variation in disease progression and treatment effect with baseline was taken into account in the analysis, it was not surprising to observe that age did not impact the analysis even though such a result could be counter-intuitive at first glance.

Table S4. Sensitivity analysis: impact of age and sex on treatment effect

|  | **Treatment compared to placebo** | | | | **Covariate interaction with treatment compared to placebo** | | | |
| --- | --- | --- | --- | --- | --- | --- | --- | --- |
|  | High-dose PXT3003 | | Low-dose PXT3003 | | High-dose PXT3003 | | Low-dose PXT3003 | |
|  | Treatment effect^a^ | p-value | Treatment effect^a^ | p-value | Treatment effect^a^ | p-value | Treatment effect^a^ | p-value |
| Age | -0.37 (-0.69, -0.06) | 0.008 | -0.13 (-0.40, 0.14) | 0.280 | -0.01 (-0.03, 0.02) | 0.598 | -0.01 (-0.03, 0.01) | 0.341 |
| Sex | -0.37 (-0.69, -0.06) | 0.008 | -0.13 (-0.40, 0.13) | 0.263 | 0.27 (-0.36, 0.90) | 0.334 | -0.34 (-0.94, 0.26) | 0.207 |

^a^ Treatment effects compared to placebo adjusted for covariates (mFAS). Values are reported as the mean effect (97.5% confidence intervals).
